# Total burden of disease in cancer patients at diagnosis—a Danish nationwide study of multimorbidity and redeemed medication

**DOI:** 10.1038/s41416-020-0950-3

**Published:** 2020-07-07

**Authors:** Katrine Loeppenthin, Susanne Oksbjerg Dalton, Christoffer Johansen, Elisabeth Andersen, Mikkel Bring Christensen, Helle Pappot, Lone Nørgaard Petersen, Lise Bjerrum Thisted, Anne Frølich, Christiane Ehlers Mortensen, Ulrik Lassen, Jytte Ørsted, Pernille Envold Bidstrup

**Affiliations:** 1 Late Effect Research Unit CASTLE, Department of Oncology, Rigshospitalet, Blegdamsvej 58, 9601, 2100 Copenhagen, Denmark; 2 Psychological Aspects of Cancer, Danish Cancer Society Research Center, Strandboulevarden 49, 2100 Copenhagen, Denmark; 3 Survivorship and Inequality in Cancer, Danish Cancer Society Research Center, Strandboulevarden 49, 2100 Copenhagen, Denmark; 4 Department of Clinical Oncology and Palliative Care, Zealand University Hospital, Naestved, Denmark; 5 Department of Oncology, Rigshospitalet, Blegdamsvej 9, 2100 Copenhagen, Denmark; 6 Statistics and Data Analysis, Danish Cancer Society Research Center, Strandboulevarden 49, 2100 Copenhagen, Denmark; 7 Department of Clinical Pharmacology, Bispebjerg and Frederiksberg Hospital, Bispebjerg Bakke 24, 2400 Copenhagen, Denmark; 8 Research Center for Multimorbidity and Chronic Conditions, Region Zealand, University of Copenhagen, Copenhagen, Denmark

**Keywords:** Cancer epidemiology, Cancer

## Abstract

**Background:**

Multimorbidity is a growing challenge worldwide. In this nationwide study, we investigated the prevalence of multimorbidity and polypharmacy at the time of diagnosis across 20 cancers.

**Methods:**

We conducted a nationwide register-based cohort study of all Danish residents with a first primary cancer diagnosed between 1 January 2005 and 31 December 2015. Multimorbidity was defined as one or more of 20 conditions (131 specific diagnoses) registered in the Danish National Patient Registry < 5 years before the cancer diagnosis. Polypharmacy was defined as five or more medications registered in the Danish National Prescription Registry and redeemed twice 2–12 months before the cancer diagnosis.

**Results:**

We included 261,745 patients with a first primary cancer, of whom 55% had at least one comorbid condition at diagnosis and 27% had two or more. The most prevalent conditions at the time of cancer diagnosis were cardiovascular disease, chronic obstructive pulmonary disease, diabetes, stroke and depression/anxiety disorder. Polypharmacy was present in one-third of the cancer patients with antihypertensives, anti-thrombotic agents, anti-hyperlipidaemic agents, analgesics and diuretics as the most prevalent redeemed medications.

**Conclusion:**

Among patients with a newly established cancer diagnosis, 55% had at least one comorbid condition and 32% were exposed to polypharmacy.

## Background

Pre-existing morbidity and polypharmacy may present a challenge for clinicians when evaluating treatment options for patients newly diagnosed with cancer. For these patients, it may be difficult to evaluate the treatment efficacy and safety of ongoing treatment, and to minimise potential drug–drug interactions between antineoplastic treatment and other medications. Multimorbidity, the presence of two or more simultaneous conditions,^1^ and polypharmacy, typically characterised by use of five drugs or more,^2–5^ are growing challenges worldwide, mainly because increasing longevity means that more people have chronic conditions. In Denmark, the reported prevalence of multimorbidity is 22% (*n* = 1,397,173),^6^ while polypharmacy is present in 33% of a Danish population (*n* = 1,424,775),^7^ and both are increasing with age. The reported prevalence of comorbidity among cancer patients varies widely, from 0.4% in a retrospective population-based cohort study of 71,148 women with breast cancer at all stages, to 81% in a population-based cohort study of 20,511 elderly patients with non-small-cell lung cancer at all stages.^8^ In previous reviews of polypharmacy in elderly cancer patients^2^ and in patients with advanced cancer,^9^ the prevalence ranged from 57% in a cross-sectional study of 385 cancer patients ≥ 70 years of age to 80% in a cross-sectional study of 117 patients > 65 years with cancer at stages I–IV.^10,11^ The observed variations in the prevalence of comorbidity and polypharmacy may be due to differences in cancer types, medications or an exclusive focus on elderly cancer patients. Furthermore, most of these studies were characterised by small samples, a cross-sectional design and were based on self-reported information. We present the first nationwide, register-based cohort study of all Danish residents with a first primary cancer where we have examined both comorbid conditions, including 131 hospital-based diagnoses, and polypharmacy, counting the use of redeemed medications across major cancer types.

## Methods

### Participants and settings

#### Linkage between population-based registries

We obtained data from the Civil Registration System (CRS),^12^ which are continuously updated with regard to vital status and residence to identify and characterise the study population. Since 1968, the CRS has assigned a unique ten-digit number, including information on date of birth and gender to all residents in Denmark, allowing for merging of data across all Danish health registries.

#### Cancer patient cohort

The cohort comprised all 261,745 Danish residents aged ≥ 18 years registered in the Danish Cancer Registry^13^ for a first primary cancer diagnosed between 1 January 2005 and 31 December 2015, who were living in Denmark 5 years before the cancer diagnosis. The Danish Cancer Registry was established in 1943 and includes diagnosis of all cancers diagnosed in Denmark. We selected the 20 most prevalent cancer diagnoses in Denmark using the International Classification of Diseases, 10th Revision (ICD-10). We combined brain cancer and central nervous system (CNS) tumours because of the small numbers of cases, leaving the following 19 groups for analysis: breast cancer (C50), lung cancer (C34), prostate cancer (C61), colon cancer (C18), rectal cancer (C19 and C20), oesophagus cancer (C15), stomach cancer (C16), oropharynx cancer (C10), liver cancer (C22), pancreas cancer (C25), bladder cancer (C67), kidney cancer (C64), uterus cancer (C54), cervix cancer (C53), ovary cancer (C56), malignant melanoma (C43), brain and CNS cancer (C70–C72), non-Hodgkin lymphoma (C85) and leukaemia (C90–C95).

#### Multimorbidity and polypharmacy

As there are no international standard definitions or measures of multimorbidity or polypharmacy, we defined them from the literature and on clinical considerations. We applied the selection of morbidities from a systematic review of 39 studies that provided explicit lists of the conditions included, ranging from 4 to 102 (mean = 18),^14^ two cross-sectional studies, one of 1,751,842 people in Scotland with 40 chronic conditions^15^ and a Danish population-based study of 1,397,173 people^16^ and, the following clinical criteria for multimorbidity:

- a chronic disease, which resulted in exclusion of acute diseases such as pneumonia and conditions considered risk factors for diseases such as hypertension and
- temporal relevance for the cancer course, thus diagnosed within 5 years of diagnosis of the first primary cancer.

Multimorbidity was defined as hospitalisation for one or more of 20 diseases according to International Classification of Diseases 10th Revision (131 specific diagnoses) (Supplementary Table 1). We identified the conditions in the Danish National Patient Registry (DNPR), which was established in 1977, and contains the diagnoses for all somatic and psychiatric inpatient hospital admissions and, from 1995, all outpatient visits.^17^ To address that conditions such as hypertension or uncomplicated diabetes are often treated by a general practitioner, and thus not included in the DNPR,^18^ we complemented, for example, diabetes diagnosis from DNPR with information on diabetic medication to obtain a more comprehensive population of persons diagnosed with diabetes.

The criteria for polypharmacy were drugs:

- with temporal importance for treatment of the cancer and thus prescribed and redeemed within 1 year of the cancer diagnosis and
- in long-term use, thus medications that were redeemed at least twice within 2–12 months before the cancer diagnosis.

We defined polypharmacy as five or more different medications,^2,3^ based on the Anatomical Therapeutic Chemical (ATC) codes, each redeemed at least twice 2–12 months before the first primary cancer diagnosis. We obtained information on redeemed drug prescriptions from the Danish National Prescription Registry,^19^ which was established in 1995 and contains information on all prescription drugs dispensed at Danish community pharmacies, including for nursing home residents.^19^ We included the following ATC codes: A, alimentary tract and metabolism (drugs for diabetes, anti-emetics and other conditions); B, blood and blood-forming organs (anti-thrombotic and other haematological drugs); C, cardiovascular system (drugs for cardiac disease, hypertension and hyperlipidaemia, and anti-diuretic drugs); D, dermatological conditions; G, genitourinary system and reproductive hormones; H, systemic hormonal preparations, except reproductive hormones and insulin; J, systemic antibiotics; L, antineoplastic and immunomodulating agents; M, musculoskeletal system (anti-inflammatory and anti-rheumatic drugs); N, nervous system (analgesics, antiepileptic and anti-parkinsonism drugs, antipsychotics and antidepressants), P, insecticides and repellents; R, respiratory system; S, sensory organs (Supplementary Table 2).

#### Statistical analyses

We determined the prevalence of multimorbidity (≥2 comorbidities within 5 years prior to their cancer diagnosis) and polypharmacy (≥5 redeemed medications within 2–12 months of cancer diagnosis) separately. We used descriptive statistics (median and interquartile (25th and 75th percentile)) to calculate the prevalence with 95% confidence intervals (CI) of multimorbidity (≥2 comorbidities) and drug prescriptions by sex (male and female), and cancer sites (breast, lung, prostate, colon, rectum, oesophagus, stomach, oropharynx, liver, pancreas, bladder, kidney, uterus, cervix, ovary, malignant melanoma, brain and cancer of the central nervous system (CNS), non-Hodgkin lymphoma and leukaemia) and according to age group at cancer diagnosis (<55, 55–69 and >70 years). We estimated the proportion of patients with multimorbidity (≥2 comorbidities) and polypharmacy (≥5 redeemed medications), respectively, based on an unadjusted logistic regression model and adjusted for age group at cancer diagnosis, cancer type and sex and the interaction between sex and cancer type. We graphically displayed our results in forest plots according to adjustment as well as gender, and in bubble plots as the percentage of each comorbidity and medication by cancer type. Finally, we calculated proportions of patients with the following combination of multimorbidity (≥2 comorbidities) and polypharmacy (≥5 redeemed medications): (i) no multimorbidity and no polypharmacy, (ii) multimorbidity and no polypharmacy, (iii) no multimorbidity and polypharmacy and (iv) multimorbidity and polypharmacy, and these were displayed as bar charts by cancer type. In supplementary analyses, we illustrate the overlap between specific comorbidities and medications with examples for three prevalent cancers: lung, liver and kidney, and these were displayed in bubble plots as the percentage of each comorbidity and medication. The analyses were performed using Stata and R statistical software.^20^

## Results

**Fig. 1:**
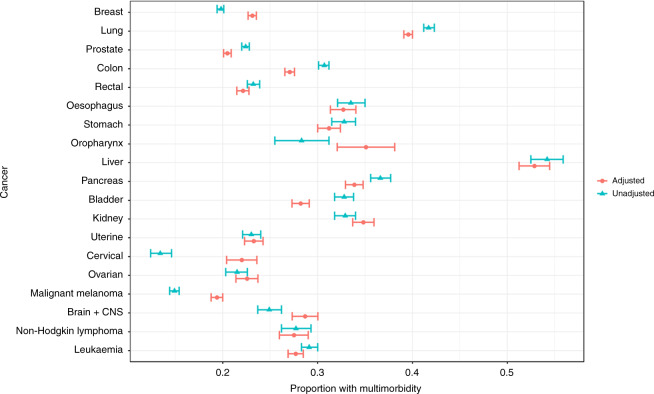
Unadjusted and adjusted (age group and sex) proportions of patients with multimorbidity (> 2 comorbidities) by cancer type among 261,745 cancer patients in the period 2005–2015, Denmark. CNS central nervous system.

**Fig. 2:**
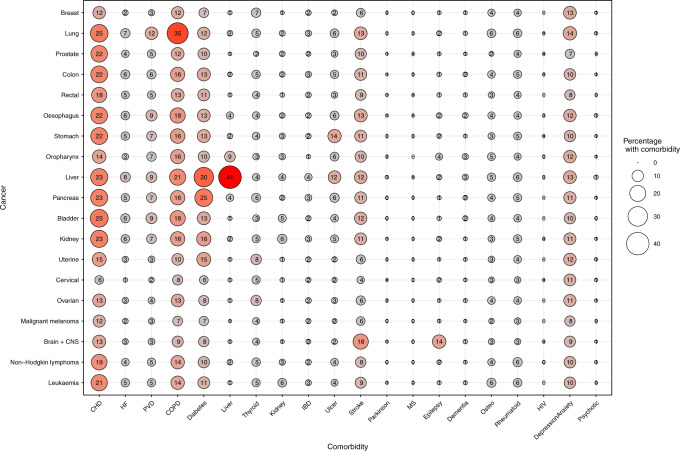
Bubble plot of the percentage of comorbidities by cancer type among 261,745 patients diagnosed in the period 2005–2015, Denmark. CI confidence interval, CHD coronary heart disease, HF heart failure, PVD peripheral vascular disease, COPD chronic obstructive pulmonary disease, Diabetes types 1 and 2, Liver liver disease, Thyroid thyroid disorders, Kidney kidney disease, IBD inflammatory bowel disease, ulcer gastric, duodenal and peptic, stroke hemiplegia, Parkinson Parkinson disease, MS multiple sclerosis, Dementia Alzheimer, vascular, Osteo osteoporosis, Rheumatoid rheumatoid arthritis, Psychotic psychotic diseases.

Of the 261,745 incident cancer patients diagnosed during the 10-year period, 55% had at least one comorbidity and 27% had at least two comorbidities (data not shown), ranging from 14% of patients with malignant melanoma to 56% of patients with liver cancer, at the time of the first primary cancer diagnosis (Table 1). Furthermore, multimorbidity (≥2 comorbidities) was most prevalent in patients aged > 70 years old across cancers where 39% had multimorbidity ranging from 29% in patients with prostate cancer to 56% in patients with liver cancer (Table 1). However, the prevalence was also high in patients aged 55–69 years across cancers, including 36% of patients with lung cancer. Comorbidity was strongly associated with increasing age, especially for patients with colon cancer, cervical cancer or malignant melanoma (Fig. 1). The presence of multimorbidity was equally distributed among females and males (Supplementary Fig. 1). The five comorbid conditions most frequently observed across cancers were cardiovascular diseases, chronic obstructive pulmonary disease, diabetes, stroke and depression/anxiety (Fig. 2).

**Table 1:** Prevalence of multimorbidity by gender, cancer type and according to age group at cancer diagnosis in 261,745 cancer patients diagnosed in the period 2005–2015, Denmark.

	Age <55 years at cancer diagnosis						Age 55–69 years at cancer diagnosis						Age ≥ 70 years at cancer diagnosis					
	Number		Comorbidity		≥2 comorbidities		Number		Comorbidity		≥2 comorbidities		Number		Comorbidity		≥2 comorbidities	
	*N*	%	Median	IQR	%	95% CI	*N*	%	Median	IQR	%	95% CI	*N*	%	Median	IQR	%	95% CI
Total	42,139		0	0–1	9	9–10	106,606		1	0–1	22	22–23	113,000		1	0–2	39	38–39
*Sex*																		
Male	12,799	30	0	0–1	11	10–11	55,381	52	1	0–1	23	22–23	61,162	54	1	0–2	38	37–38
Female	29,340	70	0	0–1	9	8–9	51,225	48	0	0–1	22	21–22	51,838	46	1	0–2	40	40–41
*Cancer site*																		
Breast	13,260	31	0	0–1	7	7–8	20,728	19	0	0–1	18	17–18	13,274	12	1	0–2	36	35–37
Lung	3517	8	0	0–1	20	19–21	16,690	16	1	0–2	36	35–36	19,608	17	2	1–3	51	50–52
Prostate	1392	3	0	0–1	8	7–10	19,680	18	0	0–1	16	15–16	21,238	19	1	0–2	29	29–30
Colon	2362	6	0	0–1	10	9–11	9174	9	0	0–1	21	20–22	15,489	14	1	0–2	40	39–40
Rectum	1675	4	0	0–1	8	7–9	6025	6	0	0–1	17	16–18	6917	6	1	0–2	33	32–34
Oesophagus	453	1	0	0–1	16	12–19	1944	2	1	0–2	29	27–31	1866	2	1	0–2	43	41–45
Stomach	688	1	0	0–1	13	10–15	2023	2	1	0–2	28	26–30	2467	2	1	0–2	43	41–45
Oropharynx	237	1	1	0–1	24	19–30	568	1	1	0–2	27	23–31	168	0.1	1	0–2	39	32–47
Liver	353	1	1	0–2	35	31–41	1455	1	2	1–3	56	54–59	1502	1	2	1–3	56	54–59
Pancreas	790	2	0	0–1	17	14–19	3444	3	1	0–2	31	29–32	4485	4	1	0–2	45	43–46
Bladder	461	1	0	0–1	14	11–17	2750	2	1	0–1	23	21–24	4923	4	1	0–2	40	39–42
Kidney	1234	3	0	0–1	16	14–19	2903	3	1	0–2	30	29–32	2415	2	1	0–2	45	43–47
Uterus	916	2	0	0–1	11	10–14	3357	3	0	0–1	17	16–18	2880	3	1	0–2	34	32–36
Cervix	2393	6	0	0–0	6	5–7	774	1	0	0–1	19	16–22	622	1	1	0–2	36	32–40
Ovary	1020	3	0	0–1	8	7–10	2019	2	0	0–1	17	16–19	1957	2	1	0–2	33	31–35
Malignant melanoma	7990	19	0	0–0	4	4–5	6049	6	0	0–1	14	14–15	4968	4	1	0–2	33	31–34
Brain + CNS	1252	3	0	0–1	11	10–13	1742	1	1	0–1	24	22–26	1367	1	1	0–2	39	36–42
Non-Hodgkin lymphoma	555	1	0	0–1	13	11–16)	1165	1	1	0–1	23	21–26	1410	1	1	0–2	37	35–40
Leukaemia	1,591	4	0	0–1	10	8–11	4116	4	1	0–1	22	21–24	5444	5	1	0–2	40	39–41

*CI* confidence interval, *CNS* central nervous system, *IQR* interquartile range.

**Fig. 3:**
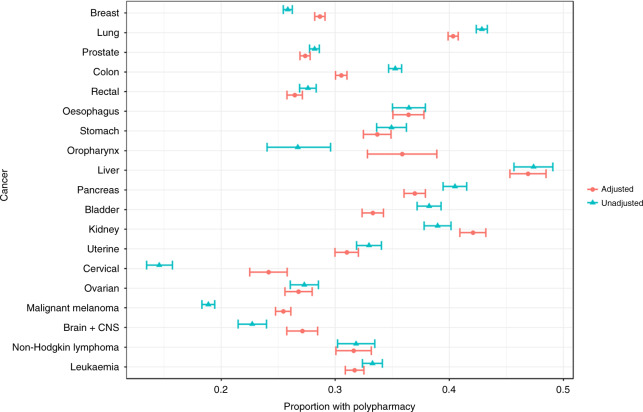
Unadjusted and adjusted (age group and sex), proportions of patients with polypharmacy (>5 medications) by cancer type among 261,745 cancer patients diagnosed in the period 2005–2015, Denmark.

**Fig. 4:**
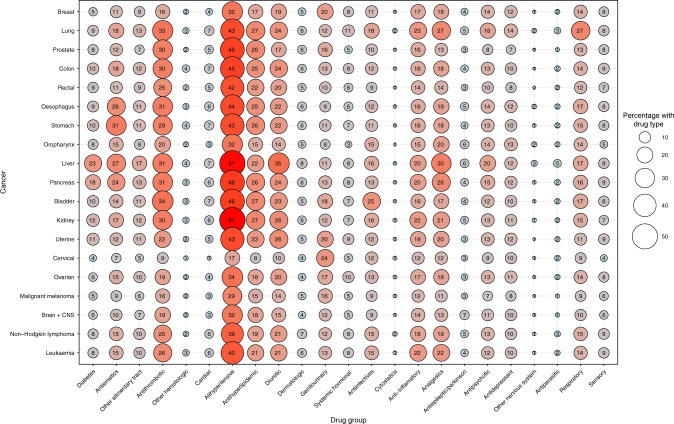
Bubble plot of the proportion of drug group by cancer type among 261,745 cancer patients diagnosed in the period 2005–2015, Denmark.

Patients used a median of three medications (interquartile range, 1–5) 2–12 months before their cancer diagnosis, and polypharmacy was present in 32% of patients across cancers and age group. As found for multimorbidity, the prevalence of polypharmacy was the highest among patients aged 55–69 years (25%) and those aged ≥ 70 years (46%) (Table 2), and increased with age at cancer diagnosis (Fig. 3). The prevalence of polypharmacy was higher in females than males (Supplementary Fig. 2). Across cancers, the five medication classes most frequently redeemed were antihypertensives, anti-thrombotic agents, anti-hyperlipidaemic agents, analgesics and diuretics (Fig. 4). Cancer patients with multimorbidity often had polypharmacy (Supplementary Fig. 1), although multimorbidity and polypharmacy were also seen independently. The cancer types with the highest proportion of both multimorbidity and polypharmacy included cancer of the lung (29%), liver (36%) and pancreas (26%) (Supplementary Fig. 3). As illustrated for three common cancer types, certain comorbidities were as expected often accompanied by certain medications; however, this also seems to vary according to cancer type (Supplementary Figs. 4–6).

**Table 2:** Prevalence of prescriptions and polypharmacy by gender, cancer type and according to age group at cancer diagnosis in 261,745 cancer patients diagnosed in the period 2005–2015, Denmark.

	Age < 55 years at cancer diagnosis						Age 55–69 years at cancer diagnosis						Age ≥70 years at cancer diagnosis					
	Number		Prescriptions		Polypharmacy		Number		Prescriptions		Polypharmacy		Number		Prescriptions		Polypharmacy	
	*N*	%	Median	IQR	%	95% CI	*N*	%	Median	IQR	%	95% CI	*N*	%	Median	IQR	%	95% CI
Total	42,139		1	0–2	9	8–9	106,606		2	0–5	25	24–26	113,000		4	2–7	46	46–47
*Sex*																		
Male	12,799	30	0	0–2	9	8–9	55,381	52	2	0–4	24	24–25	61,162	54	4	2–6	42	42–43
Female	29,340	70	1	0–2	8	8–9	51,225	48	2	1–5	26	25–26	51,838	46	5	2–8	51	51–52
*Cancer site*																		
Breast	13,260	31	1	0–2	7	7–8	20,728	19	2	0–4	23	22–24	13,274	12	4	2–7	49	48–50
Lung	3517	8	1	0–4	18	17–20	16,690	16	3	1–6	35	34–35	19,608	17	5	2–8	54	54–55
Prostate	1392	3	1	0–2	7	6–9	19,680	18	2	0–4	20	20–21	21,238	19	3	1–6	37	36–38
Colon	2362	6	1	0–2	9	8–10	9174	9	2	0–4	24	23–25	15,489	14	4	2–7	46	45–47
Rectum	1675	4	0	0–2	7	6–9	6025	6	1	0–4	19	18–20	6917	6	4	1–6	40	39–41
Oesophagus	453	1	1	0–3	13	10–16	1944	2	2	1–5	29	27–31	1866	2	4	2–7	50	48–52
Stomach	688	2	1	0–2	10	8–12	2023	2	2	1–5	28	26–30	2467	2	4	2–7	48	46–50
Oropharynx	237	1	1	0–3	18	14–24	568	1	2	0–5	26	22–30	168	0.1	4	1–7.5	42	34–49
Liver	353	1	2	0–4	25	20–29	1455	1	4	1–7	42	39–44	1502	1	5	3–8	58	56–61
Pancreas	790	2	1	0–3	14	11–16	3444	3	3	1–5.5	33	31–34	4485	4	5	2–8	51	50–53
Bladder	461	1	1	0–3	15	12–19	2750	2	2	1–5	27	26–29	4923	4	4	2–7	47	45–48
Kidney	1234	3	1	0–3	16	14–18	2903	3	3	1–6	36	34–38	2415	2	5	3–8	54	52–56
Uterus	916	2	1	0–3	15	12–17	3357	3	2	1–4	24	22–25	2880	3	4	2–7	50	48–52
Cervix	2393	6	1	0–2	5	4–6	774	1	1	0–4	21	18–24	622	1	4	2–7	43	39–47
Ovary	1020	2	1	0–2	8	6–10	2019	2	2	0–4	20	19–22	1957	2	4	2–7	45	42–47
Malignant melanoma	7990	19	0	0–1	4	4–5	6049	6	2	0–4	18	17–19	4968	4	4	2–7	44	43–45
Brain + CNS	1252	3	0	0–2	6	5–8	1742	1	1	0–4	20	18–22	1367	1	4	1–7	42	39–44
Non-Hodgkin lymphoma	555	1	1	0–2	8	6–11	1165	1	2	0–5	26	24–29	1410	1	4	2–7	46	43–48
Leukaemia	1591	4	1	0–2	8	7–10	4116	4	2	0–4	25	24–26	5444	5	4	2–7	47	46–48

*IQR* interquartile range, *CI* confidence interval.

Polypharmacy: ≥5 prescriptions within 1 year before diagnosis and at least two prescriptions. P1: test probability Kruskal–Wallis test. P2: probability chi^2^ test.

## Discussion

In this study, covering the entire population of incident cancer patients in Denmark, and including 19 cancer sites, we found that more than half of the 261,745 patients with a first primary cancer had at least one comorbidity, 27% had two or more and every third patient had five or more medications redeemed at the time of the first cancer diagnosis.

Our finding that 55% of this population of cancer patients had at least one comorbidity at diagnosis is higher than that reported in a cross-sectional study of 227,704 cancer survivors, in which it was found that 40% had a hospital-diagnosed comorbidity,^21^ but similar to the finding in a population-based sample showing that 57% of 7292 cancer survivors had self-reported comorbidity.^22^ Our study contrasts these studies in both population and methodology. Thus, the previous cross-sectional studies included cancer survivors at younger age (median age of 67 years) and a median of 6 years after cancer diagnosis,^21^ which may potentially introduce survivor bias. Furthermore, their inclusion of cancer survivors and hence patients with a favourable prognosis reduces the generalisability of the results across cancers. The observed proportion in the current study of 55% with at least one other condition is also higher than that observed in the general Danish population with 22% being multimorbid. However, the proportion that we observed of 32% with polypharmacy is similar to the reported 33% for the Danish general population with polypharmacy. The number of comorbid conditions per se may not in itself affect the cancer treatment, while the type and severity of the comorbidities probably do. Thus, patients with severe comorbidity may present with a lower performance status at diagnosis, which may be associated with ineligibility to undergo surgical treatment or impact any oncological treatment.^23^

Forty-three percent of newly diagnosed cancer patients in our study were > 70 years, and among those, 39% across cancers had multimorbidity (≥ 2 comorbidities) at the time of cancer diagnosis (ranging from 29% in prostate cancer to 56% in liver cancer). Multimorbidity may impact treatment with age, and research has, e.g., shown that elderly cancer patients (*N* = 518, >65 years) were less likely to receive systemic therapy compared with younger cancer patients, and that the treatment decisions were mainly based on patient factors, such as performance status, comorbidity, social support and cancer stage rather than age alone.^24^

We found that similar comorbid conditions were overrepresented across cancer sites. It is notable that we also found a low prevalence of Parkinson disease and multiple sclerosis, which is in line with the fact that both conditions are associated with a lower prevalence of cancer.^25,26^ In accordance with our results, it has been reported that patients with chronic obstructive pulmonary disease are at risk for later development of lung cancer,^27^ patients with repeated urinary tract infections are at risk for later development of bladder cancer^28^ and patients with gastric ulcer are at risk for later development of ventricular cancer.^29^ The mechanisms proposed to explain these phenomena include a common pathophysiology, shared environmental and lifestyle factors, a genetic predisposition, including shared genes linking to several diseases (pleiotropy) or existing disease leading to lowered robustness for development of other diseases.^30^ The cross-sectional design of our study obviates the assessment of causality between comorbidities and cancer; however, some comorbidities and cancers, e.g., of the lung, oesophagus, oropharynx, liver, bladder, breast and colon, share underlying risk factors, such as smoking, high alcohol consumption, physical inactivity and obesity.

While the prevalence of polypharmacy in our population aged > 70 years (46%) was similar to that in a cross-sectional survey of 37,959 adults aged > 65 years in the United States (39%),^31^ we also found a high prevalence (25%) in cancer patients aged 55–69 years and 9% in patients <55 years old. Although the prevalence of the specific medications varied by cancers and age groups, those most frequently prescribed were antihypertensives, anti-thrombotic, anti-inflammatory agents, analgesics and antidepressants. The high prevalence of polypharmacy, including preventive treatment with, e.g., antihypertensive and anti-hyperlipidaemic agents, specifically for patients with a cancer of known poor prognosis, such as lung cancer, does raise questions regarding the need for reviewing and prioritising among existing medications.^32^ Thus, the goal of the drug treatment to cancer patients with limited life expectancy may primarily focus on maintaining short-term quality of life and reducing treatment burden rather than preventing future diseases.

Unlike previous studies, we examined the prevalence of both multimorbidity and polypharmacy in the Danish population of cancer patients, which for the first time illustrates a large degree of overlap between multimorbidity and polypharmacy, and that certain comorbidities may be accompanied by certain prescribed medications, e.g., we observed that cardiovascular diseases, COPD, diabetes and depression/anxiety were comorbidities with a high proportion of redeemed medications. However, the proportion of medication types did seem to vary according to cancer type as illustrated by a higher proportion among patients with lung cancer compared with patients with kidney cancer. This suggests the importance of investigating both multimorbidity and polypharmacy in order to describe the potential impact on cancer patients.

With a generally increasing lifespan, a larger proportion of cancer patients are expected to present with multimorbidity and polypharmacy that may pose a high and increasing burden on the health care system in terms of costs and increased risk of hospitalisations.^6,33,34^ Previous studies have shown that patients with comorbidity have a more pronounced symptom burden,^35–37^ lower quality of life^35^ and a poorer prognosis^38^ than patients with no comorbidity. This may challenge the existing clinical guidelines for cancer treatment regimens,^39^ and call for a new paradigm of organising treatment tailored towards the individual cancer patient with complex multimorbidity and polypharmacy to guide treatment decisions.^33,40,41^

The strengths of this study include its nationwide design, covering all residents of Denmark, independent of social position. Thus, the results are generalisable to cancer patients in other affluent, industrialised countries. We used data from nationwide health registries, representing information retrieved from data sources established decades before the study hypothesis was made, and as the data were entered for administrative reasons, recall, selection and information bias are minimised. Furthermore, diagnoses in DNPR are checked for missing values, incorrect digits and inconsistencies between diagnosis and sex.^18^ In the absence of standard definitions and measures of comorbidity and polypharmacy, we have explicitly reported the comorbidities and redeemed medications included in this study. The limitations of this study include potential underestimation of certain comorbidities, as we based comorbidity mainly on hospital diagnoses, whereas some diseases, such as type 2 diabetes and arthritis, are usually diagnosed and managed in the primary health care sector.^18^ To address this, we complemented, for example, diabetes diagnosis with information on diabetic medication to obtain a more comprehensive population of persons diagnosed with diabetes. Furthermore, comorbid conditions diagnosed within the last few months of a cancer diagnosis may be more a part of the pre-diagnostic phase, representing differential diagnosis, to the cancer diagnosis rather than a comorbidity. Thus, we cannot exclude potential misclassification of comorbid conditions. Similarly, while the Danish national prescription registers only include redeemed prescriptions and hence only a surrogate measure for the actual ingestion, we only included medications that had been redeemed at least twice within 2–12 months before the cancer diagnosis in an attempt to minimise potential misclassification. Again, we may have underestimated the amount of polypharmacy, as non-prescription medications, e.g., over the counter sale of analgesics, are not included in the registry.

## Conclusion

Our finding that 55% of cancer patients had an accompanying chronic disease at the time of diagnosis, and also that 32% were exposed to polypharmacy, indicates the magnitude of the possible clinical and structural challenges of managing a large proportion of cancer patients in the future and the importance of tailored management.

## Supplementary information

Supplementary material

## Data Availability

The data are stored in a database at Statistics Denmark. According to Danish legislation, the data used in this study cannot be shared, as they are derived from nationwide registers.
