# Supplementary material for: Total burden of disease in cancer patients at diagnosis—a Danish nationwide study of multimorbidity and redeemed medication
Source: Br J Cancer. 2020 Jul 7;123(6):1033–40. doi: 10.1038/s41416-020-0950-3 (PMC7493878; doi:10.1038/s41416-020-0950-3)
Supplement: Supplementary file 1 — Supplementary material [file 41416_2020_950_MOESM1_ESM.docx]

**Supplementary material**

**Figure 1.** Estimated proportion with multimorbidity (≥ 2 comorbidities) based on logistic regression and adjusted for age group and sex.
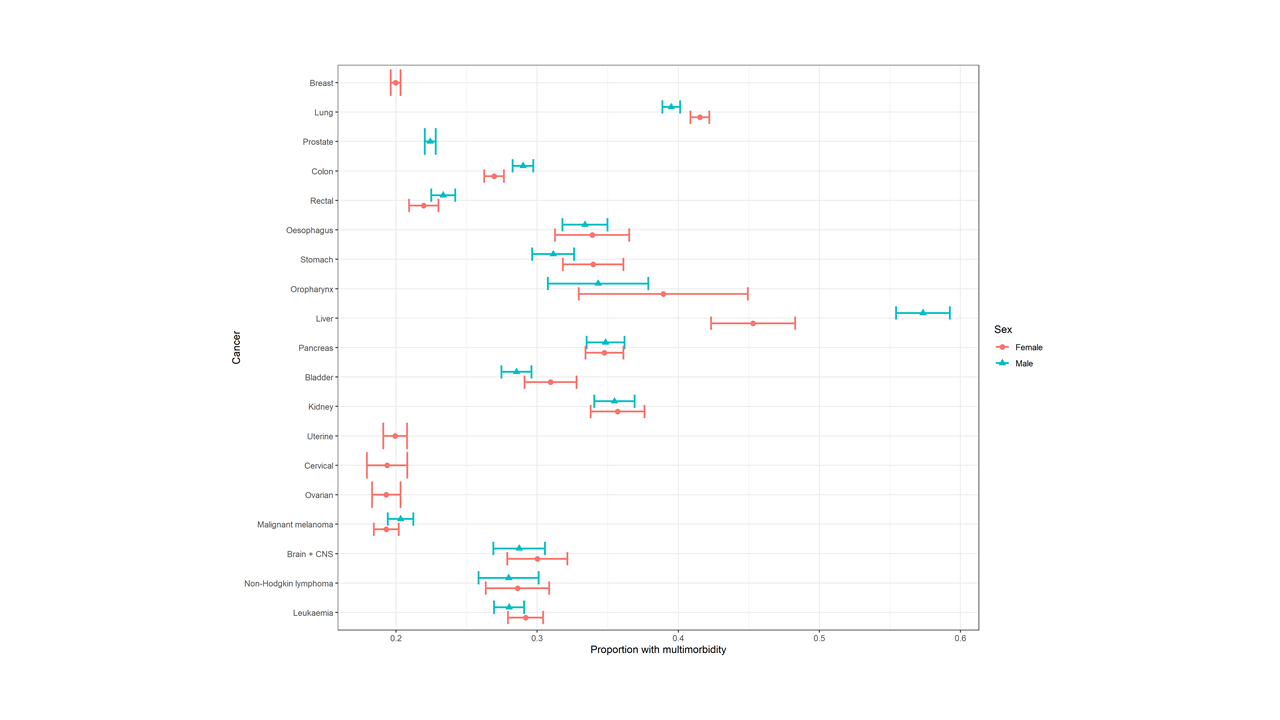


**Figure 2.** Estimated proportion with polypharmacy (≥ 5 medications) based on logistic regression and adjusted for age group and sex.
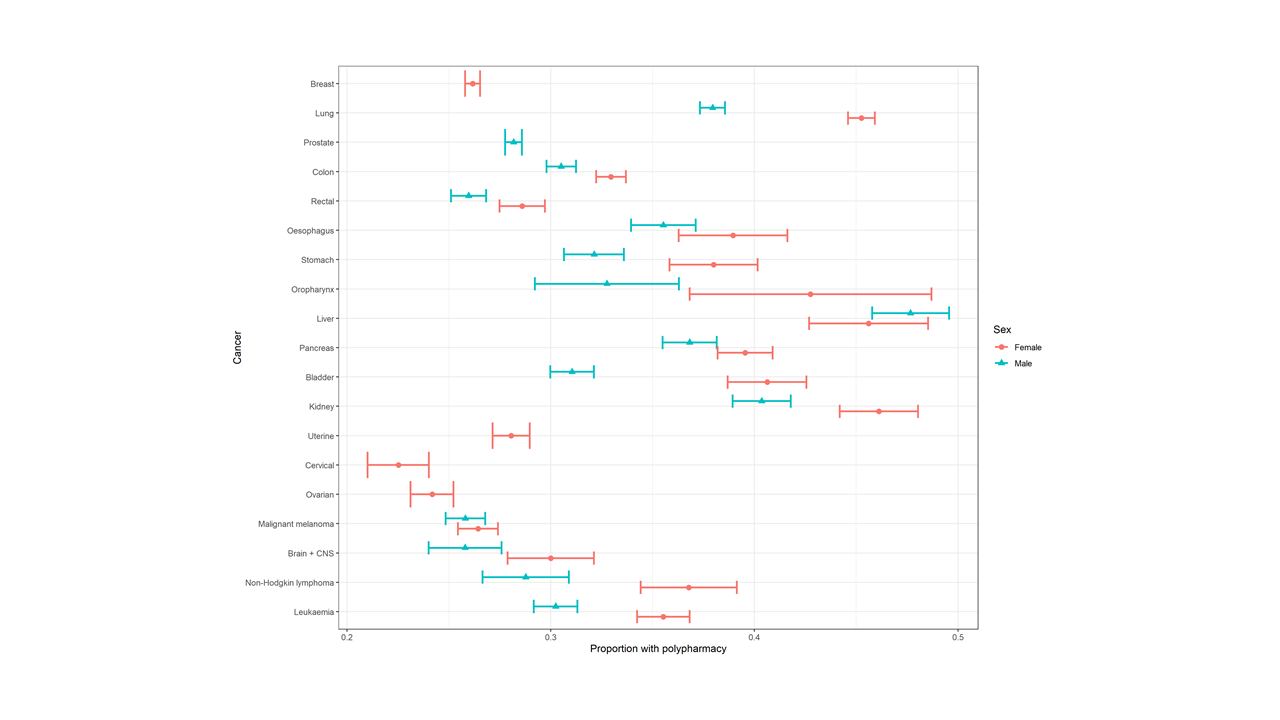


**Figure 3.** Proportion of cancer patients according to combinations of multimorbidity (≥ 2 comorbidities) and polypharmacy (≥ 5 medications) among 261,745 cancer patients diagnosed in the period 2005-2015


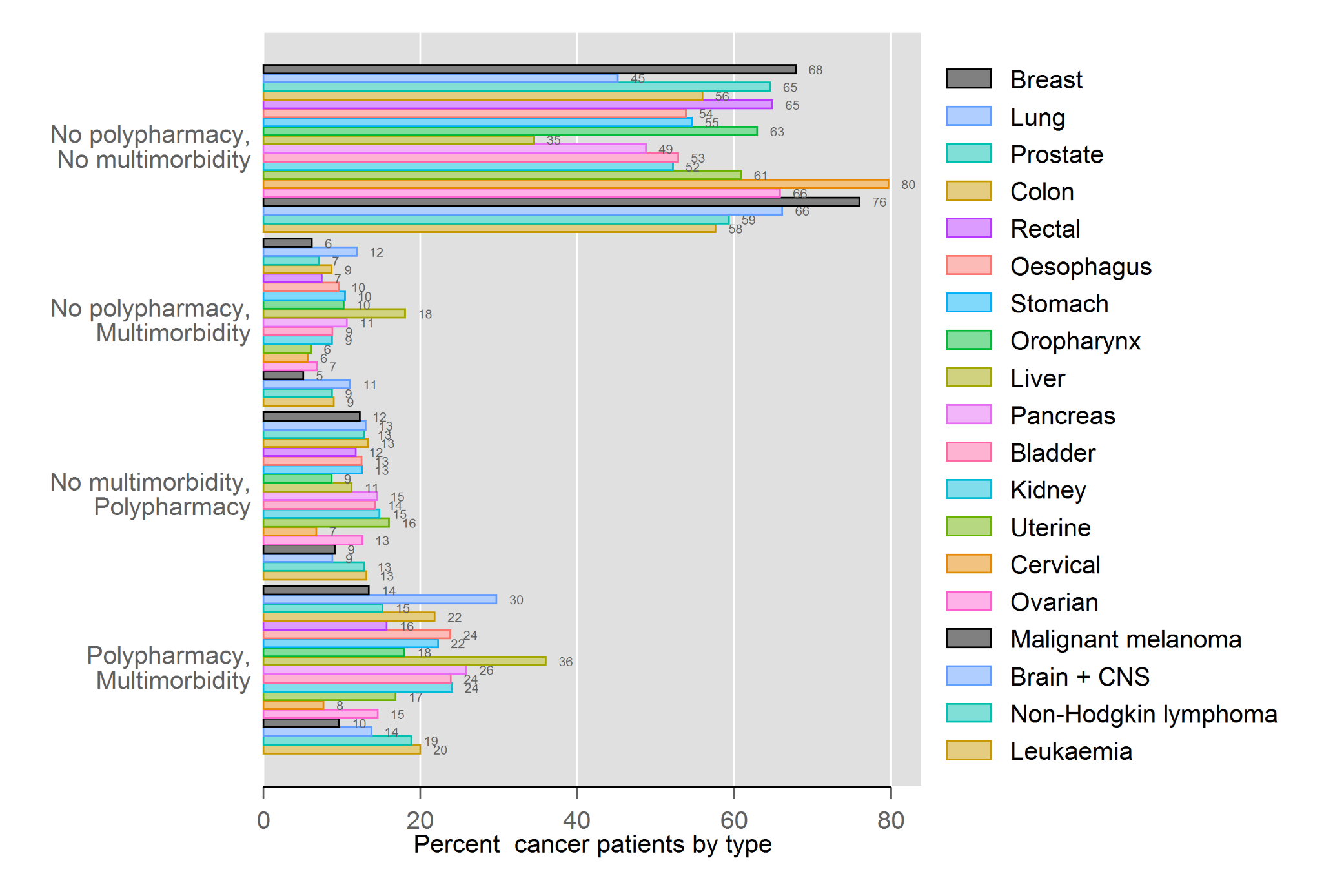


**Figure 4.** Bubble plot of proportions of patients with medication according to comorbidities in 39,815 lung cancer patients diagnosed in the period 2005-2015, Denmark


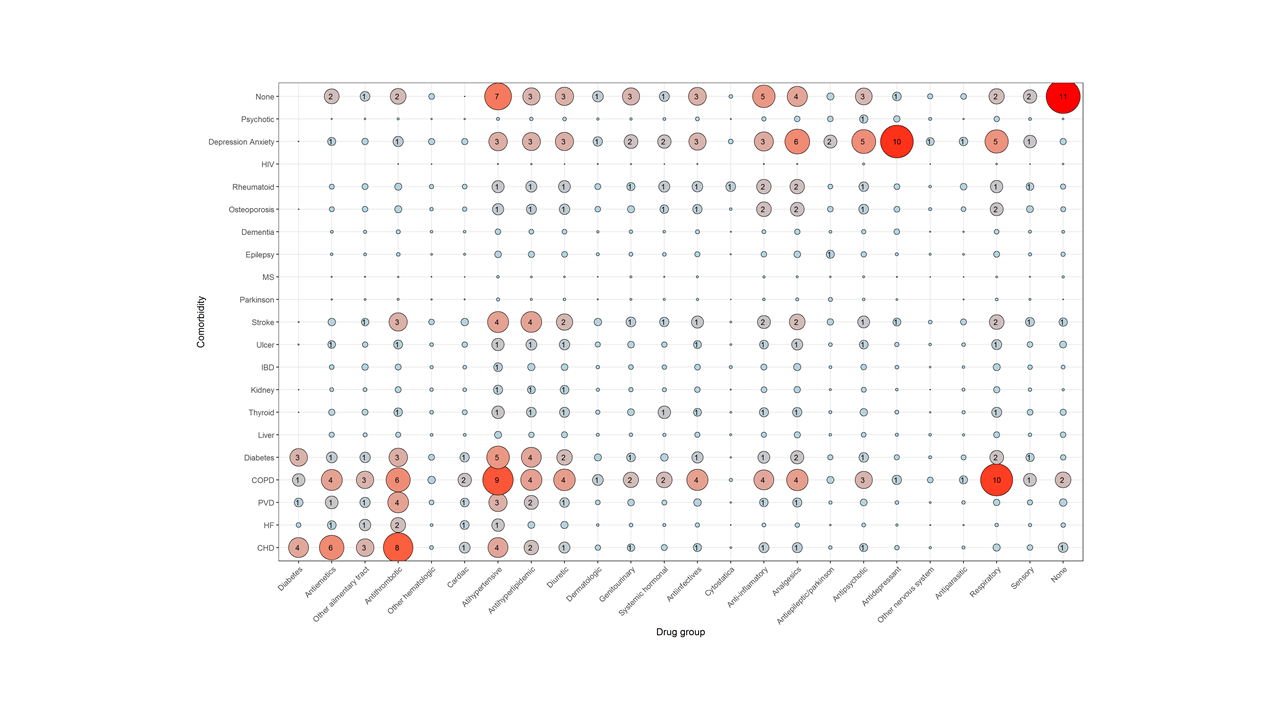


**Footnote Figure 2:** CHD, coronary heart disease; HF, heart failure; PVD, peripheral vascular disease; COPD, chronic obstructive pulmonary disease; Diabetes, types 1and 2; Liver, liver disease; Thyroid, thyroid disorders; Kidney, kidney disease; IBD, inflammatory bowel disease; Ulcer, gastric, duodenal, and peptic; Stroke, hemiplegia; Parkinson, Parkinson disease; MS, multiple sclerosis; Dementia, Alzheimer, vascular; Osteo, osteoporosis; Rheumatoid, rheumatoid arthritis; Psychotic, psychotic diseases.

**Figure 5**. Bubble plot of proportions of patients with medication according to comorbidities in 3,310 liver cancer patients

diagnosed in the period 2005-2015, Denmark


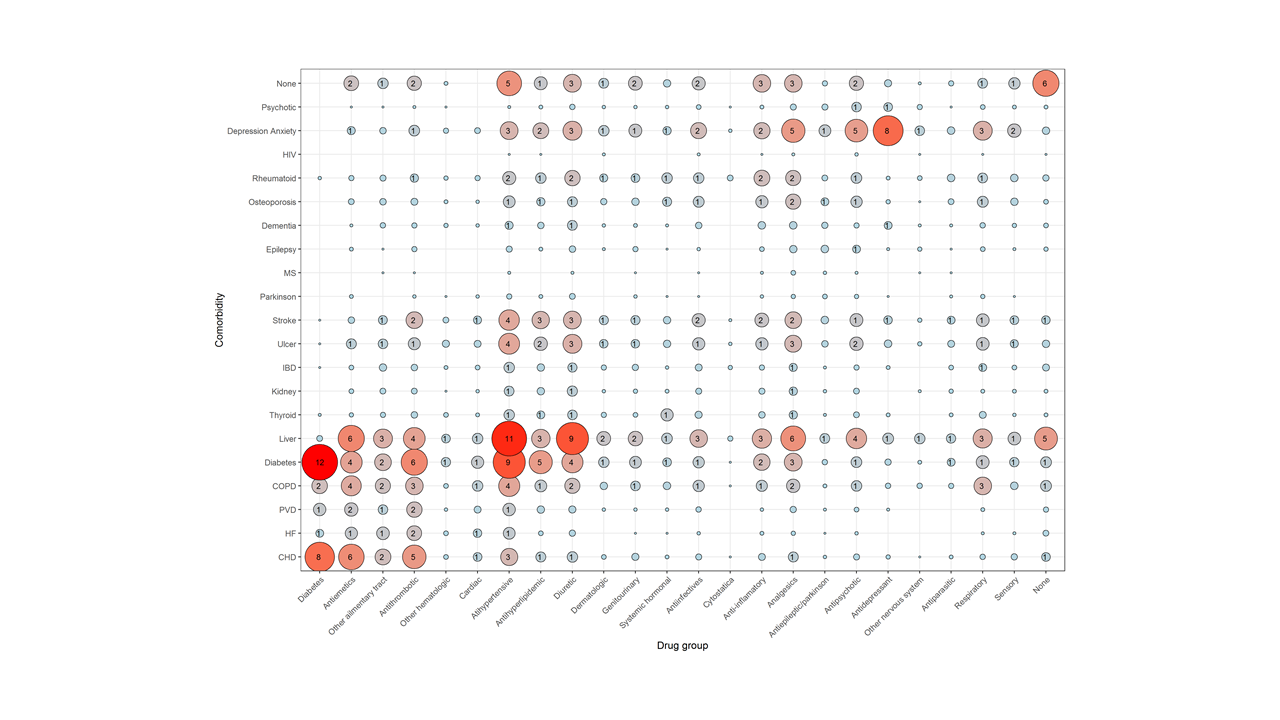


**Footnote Figure 5:** CHD, coronary heart disease; HF, heart failure; PVD, peripheral vascular disease; COPD, chronic obstructive pulmonary disease; Diabetes, types 1and 2; Liver, liver disease; Thyroid, thyroid disorders; Kidney, kidney disease; IBD, inflammatory bowel disease; Ulcer, gastric, duodenal, and peptic; Stroke, hemiplegia; Parkinson, Parkinson disease; MS, multiple sclerosis; Dementia, Alzheimer, vascular; Osteo, osteoporosis; Rheumatoid, rheumatoid arthritis; Psychotic, psychotic diseases.

**Figure 6.** Bubble plot of proportions of patients with medication according to comorbidities in 6,552 kidney cancer patients diagnosed in the period 2005-2015, Denmark


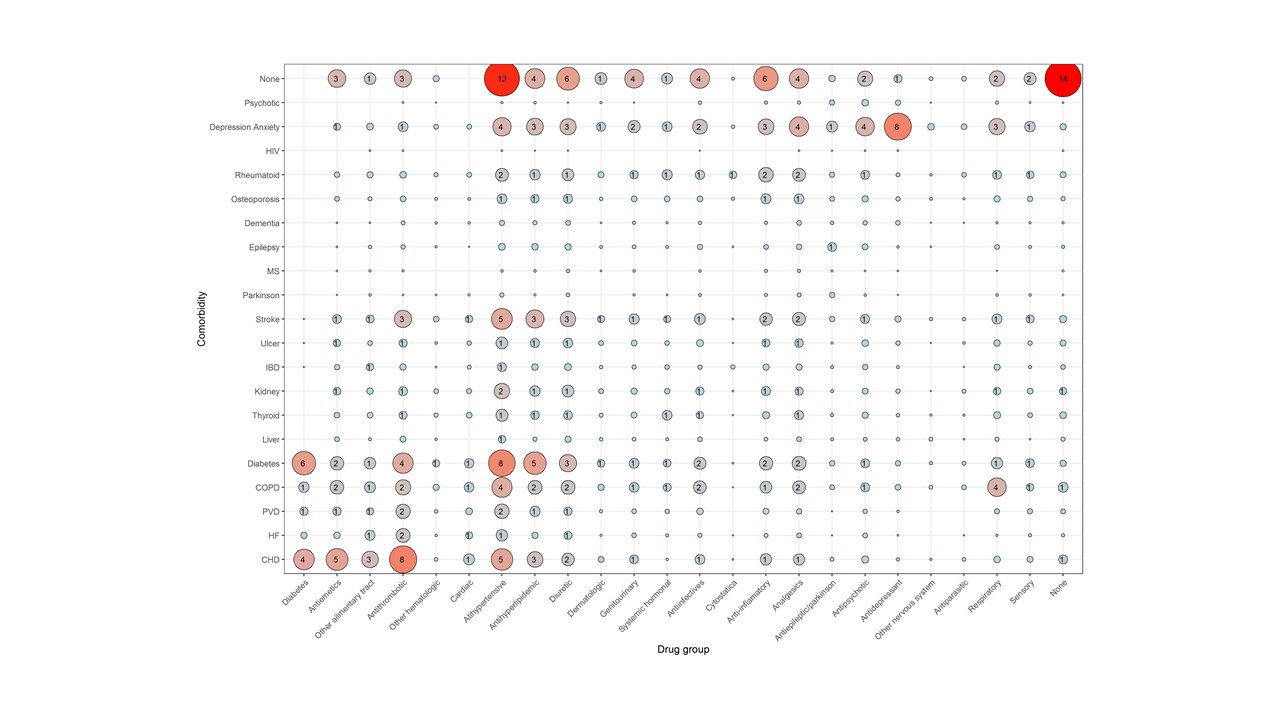


**Footnote Figure 4:** CHD, coronary heart disease; HF, heart failure; PVD, peripheral vascular disease; COPD, chronic obstructive pulmonary disease; Diabetes, types 1and 2; Liver, liver disease; Thyroid, thyroid disorders; Kidney, kidney disease; IBD, inflammatory bowel disease; Ulcer, gastric, duodenal, and peptic; Stroke, hemiplegia; Parkinson, Parkinson disease; MS, multiple sclerosis; Dementia, Alzheimer, vascular; Osteo, osteoporosis; Rheumatoid, rheumatoid arthritis; Psychotic, psychotic diseases.

# Supplementary table 1. Comorbid conditions by main disease group, ICD-10 code, specific diagnosis, and redeemed drugs

|  | Comorbidity  Main disease group | ICD-10 code | Specific diagnosis | Redeemed drugs (ATC code) |
| --- | --- | --- | --- | --- |
| 1 | Coronary heart disease | I11; I13; I20-I25; | Angina pectoris (I20), acute myocardial infarction (I21), subsequent myocardial infarction (I22), Certain current complications following acute myocardial infarction (I23), Other acute ischemic heart diseases (I24), Chronic ischemic heart disease (I25) | Diagnosis and/or medicine, all medicine prescriptions with ATCC01A, C01B, C01D, or C01E |
| 2 | Heart failure | I50 | Heart failure (I50) |  |
| 3 | Peripheral vascular disease | I70-I74; I77-I79 | Atherosclerosis (I70), aortic aneurysm and dissection (I71), other aneurysm and dissection (I72), other peripheral vascular diseases (I73), arterial embolism and thrombosis (I74), other disorders of arteries and arterioles (I77), diseases of capillaries (I78), disorders of arteries, arterioles, and capillaries in diseases classified elsewhere (I79) |  |
| 4 | Chronic obstructive pulmonary disease | J40–J44; J47; J96 | Bronchitis, not specified as acute or chronic (J40), simple and mucopurulent chronic bronchitis (J41), unspecified chronic bronchitis (J42), emphysema (J43), other chronic obstructive pulmonary disease (J44), bronchiectasis (J47), respiratory failure, not elsewhere classified (J96) | Diagnosis. All patients ≥ 35 years at contact and/or prescription of ATC R03AK, R03BB, R03CC, R03DA, or V03AN01 |
| 5 | Diabetes | E10-E14 | Type 1 diabetes mellitus (E10), type 2 diabetes mellitus (E11), malnutrition-related diabetes mellitus (E12), other specified diabetes mellitus (E13), unspecified diabetes mellitus (E14) | ATC: A10 |
| 6 | Liver disease | B18; K70–K74; K76 | Chronic viral hepatitis (B18), alcoholic liver disease (K70), toxic liver disease (K71), chronic hepatic failure (K72.1), hepatic failure, unspecified (K72.9), chronic persistent hepatitis, not elsewhere classified (K73.0), chronic lobular hepatitis, not elsewhere classified (K73.1), chronic active hepatitis, not elsewhere classified (K73.2), other chronic hepatitis, not elsewhere classified (73.8), chronic hepatitis, unspecified (K73.9), hepatic fibrosis (K74.0), hepatic sclerosis (K74.1), hepatic fibrosis with hepatic sclerosis (K74.2), primary biliary cirrhosis (K74.3), secondary biliary cirrhosis (K74.4), biliary cirrhosis unspecified (K74.5), other and unspecified cirrhosis of liver (K74.6), other diseases of liver (K76) |  |
| 7 | Thyroid disorders | E00–E07 | Congenital iodine-deficiency syndrome (E00), iodine-deficiency-related thyroid disorders and allied conditions (E01), subclinical iodine-deficiency hypothyroidism (E02), other hypothyroidism (E03), other nontoxic goitre (E04), thyrotoxicosis (hyperthyroidism) (E05), thyroiditis (E06), other disorders of thyroid (E07) |  |
| 8 | Kidney disease | I12; N00-N05; N11; N18-N19; Q61 | Hypertensive renal disease (I12), acute nephritic syndrome (N00), rapidly progressive nephritic syndrome (N01), recurrent and persistent haematuria (N03), nephrotic syndrome (N04), unspecified nephritic syndrome (N05), chronic tumulo-interstitial nephritis (N11), chronic kidney disease (N18), unspecified kidney failure (N19), cystic kidney disease (Q61) |  |
| 9 | Inflammatory bowel disease | K50-K52 | Crohn disease (K50), ulcerative colitis (K51), other noninfective gastroenteritis and colitis (K52) |  |
| 10 | Ulcer | K25-K28 | Gastric ulcer (K25), duodenal ulcer (K26), peptic ulcer, site unspecified (K27), gastrojejunal ulcer (K28) |  |
| 11 | Hemiplegia/stroke | G81; G82; G45, G46; I60–I69, | Hemiplegia (G81), paraplegia and tetraplegia (G82), transient cerebral ischaemic attacks and related syndromes (G45), vascular syndromes of brain in cerebrovascular diseases (G46), cerebrovascular diseases (I60–I69) |  |
| 12 | Parkinson disease | G20–G22 | Parkinson disease (G20), secondary parkinsonism (G21), parkinsonism in diseases classified elsewhere (G22) |  |
| 13 | Multiple sclerosis | G35 | Multiple sclerosis (G35) |  |
| 14 | Epilepsy | G40-G41 | Epilepsy (G40), status epilepticus (G41) |  |
| 15 | Dementia | F00–F03; F05.1; G30–G31 | Dementia in Alzheimer disease (F00), vascular dementia (F01), dementia in other diseases classified elsewhere (F02), unspecified dementia (F03), Alzheimer disease (G30), other degenerative diseases of nervous system, not elsewhere classified (G31) | All medicines with the prescription ATC N06D |
| 16 | Osteoporosis | M80–M82 and for people aged ≥ 45 years and contacts with ICD-10 codes S22.0, S32.0 | Postmenopausal osteoporosis with pathological fracture (M80.0), post-oophorectomy osteoporosis with pathological fracture (M80.1), osteoporosis of disuse with pathological fracture (80.2), postsurgical malabsorption osteoporosis with pathological fracture (M80.3), drug-induced osteoporosis with pathological fracture (M80.4), idiopathic osteoporosis with pathological fracture (M80.5), other osteoporosis with pathological fracture (80.8), unspecified osteoporosis with pathological fracture (M80.9), osteoporosis without pathological fracture (M81), osteoporosis in diseases classified elsewhere (M82), fracture of rib(s), sternum or thoracic spine (S22), fracture of lumbar spine or pelvis (S32), | Diagnosis and/or medicine, all medicine prescriptions with ATC M05B, G03XC01, H05AA02, or H05AA03 |
| 17 | Rheumatoid and connective tissue | M05; M06; M07, M08; M09; M10, M10.9, M30–M36 | Seropositive rheumatoid arthritis (M05); other rheumatoid arthritis (M06); other soft tissue disorders (M07); juvenile arthritis (M08); juvenile arthritis in diseases classified elsewhere (M09); systemic connective tissue disorders (M30-M36) |  |
| 18 | HIV/AIDS | B20-B24 | HIV disease resulting in infectious and parasitic diseases (B20), HIV diseases resulting in malignant neoplasms (B21), HIV disease resulting in other specified diseases (B22), HIV disease resulting in other conditions (B23), unspecified HIV disease (B24) |  |
| 19 | Depression, anxiety, and other neurotic, stress related and somatoform disorders | F32–F34  F40–F45, F48 | Depressive episode (F32), recurrent depressive disorder (F33), persistent mood (affective) disorders (F34), phobic anxiety disorders (F40), other anxiety disorders (F41), obsessive­–compulsive disorder (F42), reaction to severe stress and adjustment disorders (F43), dissociative (conversion) disorders (F44), somatoform disorders (F45), other neurotic disorders (F48) | Diagnosis (F32–F34 or F40–F45, F48) and/or at least three prescriptions for ATC N06A [At least three prescriptions on different dates within the latest 5 years with at least 730 days (2 years) between the first and the last one] |
| 20 | Psychotic diseases | F20–F25, F28–F31 | Schizophrenia (F20), schizotypal disorder (F21), persistent delusional disorders (F22), acute and transient psychotic disorders (F23), induced delusional disorder (F24), schizoaffective disorders (F25), other nonorganic psychotic disorders (F28), unspecified nonorganic psychosis (F29), manic episode (F30), bipolar affective disorder (F31) |  |

ICD-10, International Classification of Diseases 10th Revision; ATC, Anatomical Therapeutic Chemical codes

# Supplementary material

# Supplementary Table 2. Groups of medication by main group, ATC code, and drug name

|  | **Main group** | **ATC** | **Drug** |
| --- | --- | --- | --- |
| A | [Alimentary tract and metabolism](http://www.atccode.com/A) | **Drugs for diabetes** | |
|  |  | A10 | [Drugs used in diabetes](http://www.atccode.com/A10) |
|  |  | **Antiemetics** | |
|  |  | A01A | [Stomatological preparations](http://www.atccode.com/A01A) |
|  |  | A02 | [Drugs for acid-related disorders](http://www.atccode.com/A02) |
|  |  | A03 | [Drugs for functional gastrointestinal disorders](http://www.atccode.com/A03) |
|  |  | A04A | [Antiemetics and antinauseants](http://www.atccode.com/A04A) |
|  |  | **Other alimentary tract and metabolism products** | |
|  |  | A05 | [Bile and liver therapy](http://www.atccode.com/A05) |
|  |  | A06A | [Drugs for constipation](http://www.atccode.com/A06A) |
|  |  | A07 | [Antidiarrheals, intestinal anti-inflammatory/anti-infective agents](http://www.atccode.com/A07) |
|  |  | A08 | [Antiobesity preparations, excluding diet products](http://www.atccode.com/A08) |
|  |  | A09A | [Digestives, including enzymes](http://www.atccode.com/A09A) |
|  |  | A15 | Appetite stimulants |
|  |  | A16A | [Other alimentary tract and metabolism products](http://www.atccode.com/A16A) |
|  | | | |
| B | [Blood and blood-forming organs](http://www.atccode.com/B) | **Antithrombotic drugs** | |
|  |  | B01A | [Antithrombotic agents](http://www.atccode.com/B01A) |
|  |  | B02 | [Antihaemorrhagic drugs](http://www.atccode.com/B02) |
|  |  | **Other haematological drugs** | |
|  |  | B03 | [Antianaemic drugs](http://www.atccode.com/B03) |
|  |  | B05 | [Blood substitutes and perfusion solutions](http://www.atccode.com/B05) |
|  |  | B06A | [Other haematological drugs](http://www.atccode.com/B06A) |
|  | | | |
| C | [Cardiovascular system](http://www.atccode.com/C) | **Cardiac therapy** | |
|  |  | C01 | [Cardiac therapy](http://www.atccode.com/C01) |
|  |  | **Antihypertensive drugs** | |
|  |  | C02 | [Antihypertensive drugs](http://www.atccode.com/C02) |
|  |  | C04 | [Peripheral vasodilators](http://www.atccode.com/C04) |
|  |  | C05 | [Vasoprotective drugs](http://www.atccode.com/C05) |
|  |  | C07 | [Beta-blocking agents](http://www.atccode.com/C07) |
|  |  | C08 | [Calcium channel blockers](http://www.atccode.com/C08) |
|  |  | C09 | [Agents that act on the renin–angiotensin system](http://www.atccode.com/C09) |
|  |  | **Antihyperlipidemic agents** | |
|  |  | C10 | [Lipid-modifying agents](http://www.atccode.com/C10) |
|  |  | **Diuretic** | |
|  |  | C03 | [Diuretic drugs](http://www.atccode.com/C03) |
|  | | | |
| D | [Dermatological drugs](http://www.atccode.com/D) | Dermatological drugs | |
|  |  | D01 | [Antifungals for dermatological use](http://www.atccode.com/D01) |
|  |  | D02 | [Emollients and protectants](http://www.atccode.com/D02) |
|  |  | D03 | [Treatment of wounds and ulcers](http://www.atccode.com/D03) |
|  |  | D04 | [Antipyretics drugs](http://www.atccode.com/D04) |
|  |  | D05 | [Antipsoriatic drugs](http://www.atccode.com/D05) |
|  |  | D06 | [Antibiotics and chemotherapeutics for dermatological use](http://www.atccode.com/D06) |
|  |  | D07 | [Topical dermatological corticosteroids](http://www.atccode.com/D07) |
|  |  | D08 | [Antiseptics and disinfectants drugs](http://www.atccode.com/D08) |
|  |  | D09 | [Medicated dressings](http://www.atccode.com/D09) |
|  |  | D10 | [Acne drugs](http://www.atccode.com/D10) |
|  |  | D11 | [Other dermatological drugs](http://www.atccode.com/D11) |
|  | | | |
| G | [Genitourinary system and reproductive hormones](http://www.atccode.com/G) | G01 | [Gynaecological anti-infectives and antiseptics](http://www.atccode.com/G01) |
|  |  | G02 | [Other gynaecological drugs](http://www.atccode.com/G02) |
|  |  | G03 | [Sex hormones and modulators of the genital system](http://www.atccode.com/G03) |
|  |  | G04 | [Urological drugs](http://www.atccode.com/G04) |
|  | | | |
| H | [Systemic hormonal preparations, excluding reproductive hormones and insulins](http://www.atccode.com/H) | H01 | [Pituitary and hypothalamic hormones and analogues](http://www.atccode.com/H01) |
|  |  | H02 | [Corticosteroids, systemic](http://www.atccode.com/H02) |
|  |  | H03 | [Thyroid therapy](http://www.atccode.com/H03) |
|  |  | H04 | [Pancreatic hormones](http://www.atccode.com/H04) |
|  |  | H05 | [Calcium homeostasis](http://www.atccode.com/H05) |
|  |  |  |  |
| J | [Anti-infectives for systemic use](http://www.atccode.com/J) | J01 | [Antibacterial drugs](http://www.atccode.com/J01) |
|  |  | J02 | [Antimycotic drugs](http://www.atccode.com/J02) |
|  |  | J04 | [Antimycobacterials](http://www.atccode.com/J04) |
|  |  | J05 | [Antiviral drugs](http://www.atccode.com/J05) |
|  |  | J06 | [Immune sera and immunoglobulins](http://www.atccode.com/J06) |
|  |  | J07 | [Vaccines](http://www.atccode.com/J07) |
|  |  |  |  |
| L | [Antineoplastic and immunomodulating agents](http://www.atccode.com/L) | Cytostatics | |
|  |  | L01 | [Antineoplastic drugs](http://www.atccode.com/L01) |
|  |  | L02 | [Endocrine therapy](http://www.atccode.com/L02) |
|  |  | L03A | [Immunostimulant drugs](http://www.atccode.com/L03A) |
|  |  | L04A | [Immunosuppressant drugs](http://www.atccode.com/L04A) |
|  | | | |
| M | [Musculoskeletal system](http://www.atccode.com/M) | **Anti-inflammatory and anti-rheumatic drugs** | |
|  |  | M01 | [Anti-inflammatory and antirheumatic drugs](http://www.atccode.com/M01) |
|  |  | M02 | [Topical products for joint and muscular pain](http://www.atccode.com/M02) |
|  |  | M03 | [Muscle relaxants](http://www.atccode.com/M03) |
|  |  | M04A | [Antigout preparations](http://www.atccode.com/M04A) |
|  |  | M05B | [Drugs that affect bone structure and mineralisation](http://www.atccode.com/M05B) |
|  |  | M09A | [Other drugs for disorders of the musculoskeletal system](http://www.atccode.com/M09A) |
|  | | | |
| N | [Nervous system](http://www.atccode.com/N) |  |  |
|  |  | **Analgesics** | |
|  |  | N02 | [Analgesic drugs](http://www.atccode.com/N02) |
|  |  | **Antiepileptic or anti-parkinsonism drugs** | |
|  |  | N03A | [Antiepileptic drugs](http://www.atccode.com/N03A) |
|  |  | N04 | [Anti-parkinsonism drugs](http://www.atccode.com/N04) |
|  |  | **Antipsychotic drugs** | |
|  |  | N05 | [Psycholeptics](http://www.atccode.com/N05) |
|  |  | **Antidepressants** | |
|  |  | N06 | [Psycho-analeptics](http://www.atccode.com/N06) |
|  |  | **Other nervous system drugs** | |
|  |  | N01 | [Anaesthetic drugs](http://www.atccode.com/N01) |
|  |  | N07 | [Other nervous system drugs](http://www.atccode.com/N07) |
|  | | | |
| P | [Antiparasitic products, insecticides and repellents](http://www.atccode.com/P) | P01 | [Antiprotozoal drugs](http://www.atccode.com/P01) |
|  |  | P02 | [Anthelmintic drugs](http://www.atccode.com/P02) |
|  |  | P03 | [Ectoparasiticides, including scabicides, insecticides, and repellents](http://www.atccode.com/P03) |
|  | | | |
| R | [Respiratory system](http://www.atccode.com/R) | R01 | [Nasal preparations](http://www.atccode.com/R01) |
|  |  | R02A | [Throat drugs](http://www.atccode.com/R02A) |
|  |  | R03 | [Drugs for obstructive airway diseases](http://www.atccode.com/R03) |
|  |  | R05 | [Cough and cold drugs](http://www.atccode.com/R05) |
|  |  | R06A | [Antihistamines for systemic use](http://www.atccode.com/R06A) |
|  |  | R07A | [Other respiratory system products](http://www.atccode.com/R07A) |
|  | | | |
| S | [Sensory organs](http://www.atccode.com/S) | S01 | [Ophthalmological drugs](http://www.atccode.com/S01) |
|  |  | S02 | [Otologicals](http://www.atccode.com/S02) |
|  |  | S03 | [Ophthalmological and otological preparations](http://www.atccode.com/S03) |
|  |  |  |  |
|  |  |  |  |
| V | [Various ATC structures](http://www.atccode.com/V) | V01AA | [Allergen extracts](http://www.atccode.com/V01AA) |
|  |  | V03A | [All other therapeutic products](http://www.atccode.com/V03A) |
|  |  | V04 | [Diagnostic agents](http://www.atccode.com/V04) |
|  |  | V06 | [General nutrients](http://www.atccode.com/V06) |
|  |  | V07 | All other non-therapeutic products |
|  |  | V08 | [Contrast media](http://www.atccode.com/V08) |
|  |  | V09 | [Diagnostic radiopharmaceuticals](http://www.atccode.com/V09) |
|  |  | V10 | [Therapeutic radiopharmaceuticals](http://www.atccode.com/V10) |
|  |  | V20 | Surgical dressings |

ATC, Anatomical Therapeutic Chemical code
